# New Features Using Robust MVDR Spectrum of Filtered Autocorrelation Sequence for Robust Speech Recognition

**DOI:** 10.1155/2013/634160

**Published:** 2013-12-31

**Authors:** Sanaz Seyedin, Seyed Mohammad Ahadi, Saeed Gazor

**Affiliations:** ^1^Department of Electrical and Computer Engineering, Queen's University, Kingston, ON, Canada K7L 3N6; ^2^Department of Electrical Engineering, Amirkabir University of Technology, Tehran 15914, Iran

## Abstract

This paper presents a novel noise-robust feature extraction method for speech recognition using the robust perceptual minimum variance distortionless response (MVDR) spectrum of temporally filtered autocorrelation sequence. The perceptual MVDR spectrum of the filtered short-time autocorrelation sequence can reduce the effects of residue of the nonstationary additive noise which remains after filtering the autocorrelation. To achieve a more robust front-end, we also modify the robust distortionless constraint of the MVDR spectral estimation method via revised weighting of the subband power spectrum values based on the sub-band signal to noise ratios (SNRs), which adjusts it to the new proposed approach. This new function allows the components of the input signal at the frequencies least affected by noise to pass with larger weights and attenuates more effectively the noisy and undesired components. This modification results in reduction of the noise residuals of the estimated spectrum from the filtered autocorrelation sequence, thereby leading to a more robust algorithm. Our proposed method, when evaluated on Aurora 2 task for recognition purposes, outperformed all Mel frequency cepstral coefficients (MFCC) as the baseline, relative autocorrelation sequence MFCC (RAS-MFCC), and the MVDR-based features in several different noisy conditions.

## 1. Introduction

Speech recognition systems are usually trained in clean conditions and tested in different environments (clean and noisy). This mismatch between the training and test conditions drastically degrades the performance of automatic speech recognition (ASR) systems in noisy environments. Robust speech recognition is considered as one of the most challenging areas in speech processing technology since the type of the noise encountered in test conditions is usually not predictable. Robust speech recognition methods may be classified into four main categories [1]:

1. robust speech feature extraction,
2. speech enhancement for improved recognition,
3. model-based compensation for noise,
4. model-based feature enhancement.

Finding a set of parameters which are robust against the variations made by different noises on speech signals is the main purpose of the first method. This category, itself, can be further classified into two main divisions:

1. extracting more robust features,
2. postprocessing of the features for robustness.

Many speech processing systems such as speech enhancement, speech recognition, and speech coding use the magnitude information of speech signals in some sparse domain such as short time fourier transform (STFT) [2, 3]. For instance, mel frequency cepstral coefficients (MFCC) [2], and perceptual linear prediction (PLP) [3] are considered as famous features used. Therefore, modifying the power spectrum of the speech signal to make it robust against additive or convolutional distortions is more widely used in the former type. Among the useful methods in this category, we can refer to differential power spectrum (DPS) [4], autocorrelation Mel frequency cepstral coefficients (AMFCC) [5], relative autocorrelation sequence MFCC (RAS-MFCC) [6], differentiated autocorrelation sequence (DAS) [7], and DCT and MVDR-based features [8–11]. Feature extraction algorithms based on auditory system such as power normalized cepstral coefficients (PNCC) [12] and Fourier-Bessel Cepstral Coefficients (FBCC) [13] are also among other methods in the former group. Enhancing Mel-filtered log spectrum of noisy signals based on the estimated distribution of speech in this domain has also been proposed for extracting more robust features [14]. Furthermore, feature normalization is considered as one of the most significant group of methods in the postprocessing of features. Histogram equalization (HEQ) [15], cepstral moment normalization methods [16, 17], and cepstral subband normalization (CSN) [18] are the best examples in this division.

In this paper, we aim to modify the power spectrum of the noisy speech signal to obtain more robust features. This method is categorized in the first division of robust feature extraction strategies. In the proposed approach, we extract robust MVDR spectrum of filtered autocorrelation sequence. Therefore, the robustness in our method is achieved due to the following approaches:

1. filtering the short-time autocorrelation sequence, in order to reduce the noise effects,
2. extracting the MVDR spectrum, instead of the common periodogram technique,
3. improving the MVDR spectrum and obtaining a more robust one, to reduce the residual noise effects, even for nonstationary noises.

Spectral estimation methods are either nonparametric or parametric [19]. While the FFT-based periodogram is the most popular method of the former strategy, especially in speech recognition areas, model identification and MVDR methods are among the most well-known approaches of the latter [19]. The model identification methods are classified into three divisions, namely, auto regressive (AR), moving average (MA), and ARMA [19]. The traditional speech features, namely, MFCC are extracted from the FFT-based periodogram power spectrum, whose estimation suffers from large bias and variance [10]. Bias is mainly caused by the leakage of power from surrounding frequencies of the band-pass filter used to measure the power [10]. Large variance is due to employing a single sample in the power estimation process [10]. Both of these shortcomings have been addressed by the MVDR spectrum estimation method [8, 10]. Incorporating MVDR spectral estimation method which reduces the bias and variance of the spectrum estimates would be effective in extracting robust speech features. This happens because (i) the bias and variance of the spectrum estimate affect speech features which are used to extract Gaussian parameters modeling the speech classes, (ii) increasing the level of noise in noisy signals enlarges the variance of the power spectrum and thus deteriorates the recognition accuracy, and (iii) other widely used features in ASR systems, namely, linear prediction (LP) [2] and PLP, which are based on AR or LP methods, are not good candidates for accurate estimation of the power spectrum of voiced speech, especially high-pitch voices. Thus, the LP-based spectrum will also be sensitive to noise, because its envelope tends to follow the fine structure of speech spectrum in such voices [10]. Therefore, we suggest using MVDR-based speech feature extraction as an appropriate approach for making ASR systems more robust against noise.

In addition, filtering the temporal trajectories of short-time autocorrelation sequences, denoted as RAS, is helpful in removing the noise effects in case of stationary noise [6]. However, the additive noise encountered in most ASR systems is nonstationary, and therefore this technique cannot remove the distortions completely. For this reason, we propose to find the MVDR spectrum of this filtered autocorrelation sequence to further reduce the noise residuals. Moreover, in order to make the proposed method more robust even in low signal to noise ratios (SNRs), we suggest using a robust MVDR approach similar to [11]. The robustness in [11] is achieved by modifying the distortionless constraint of the MVDR spectral estimation method by weighting the subband power spectrum values based on the subband SNRs. In this paper, we also modify the weighting function proposed in [11] to not only adjust it to the new proposed approach but also improve the recognition accuracies in both high and low SNRs. We have suggested this modification on the weighting function to adapt it to the proposed procedure on the perceptual spectrum of temporally filtered autocorrelation sequence, which has higher subband SNR compared to non-filtered case. The higher subband SNR is caused by suppressing some parts of the noise by the mentioned temporal filter. This new function which introduces a new robust distortionless constraint for the filtered MVDR spectrum causes more reliable components of the input signal at the frequencies least affected by noise to pass with larger weights, while attenuating the noisy (less reliable) components with smaller weights. Hence, the noise effects still remained after applying the mentioned filter on the autocorrelation sequence will be reduced, which is helpful especially for non-stationary noise. The recognition results show that this strategy is very helpful in extracting more robust features.

The paper is organized as follows. In Section 2, we briefly describe the robust MVDR spectral estimation as the related work. Our proposed robust front-end is given in Section 3. The experimental results are presented in Section 4. Finally, discussion and conclusions are given in Sections 5 and 6, respectively.

## 2. Related Work: Robust MVDR Spectral Estimation

The main purpose of MVDR spectral estimation is reducing the bias and variance of the estimated spectrum. This goal is achieved by designing an FIR filter, *h*(*n*), which minimizes its output power subject to the constraint that its response at the frequency of interest, *ω*_*l*_, has unity gain. This distortionless constraint certifies that the components of the input signal with the frequency of interest pass without any distortion through the filter. Moreover, the output power minimization precludes the leakage of power from surrounding frequencies, resulting in reduced bias. The power of signal at the frequency of interest will be equal to the power of the filtered signal [10, 19]. Hence, computing the power spectrum using all of the output samples decreases the variance. The MVDR filter is designed by solving the following constrained optimization problem [10]:

(1) $$\begin{array}{c} \underset{h}{\min}\;h^{H}R_{L+1}h\;\text{subject}\;\text{to}\;\;v^{H}\left(w_{l}\right)h=1 \end{array}$$

which results in

(2) $$\begin{array}{c} h_{l}=\frac{R_{L+1}^{-1}v\left(\omega_{l}\right)}{v^{H}\left(\omega_{l}\right)R_{L+1}^{-1}v\left(\omega_{l}\right)}, \end{array}$$

where **v**(*ω*) = [1, *e* ^*jω*^, *e* ^*j*2*ω*^,…, *e* ^*jLω*^]  , **R**_*L*+1_  is the (*L* + 1)×(*L* + 1) Toeplitz autocorrelation matrix of the data, and **h** = [*h*_0_, *h*_1_,…, *h*_*L*_]^*T*^  . The MVDR spectrum for all of the frequencies is then computed by [10]

(3) $$\begin{array}{c} P_{\text{MV}}\left(\omega \right)=\frac{1}{v^{H}\left(\omega \right)R_{L+1}^{-1}v\left(\omega \right)}. \end{array}$$

According to the distortionless constraint in (1), the filter responses at all frequencies contribute to the final result with the same weighting since all have unity gains. However, noise usually affects the speech signal differently in various frequencies. Consequently, if some frequencies are corrupted by noise, the resulting MVDR power spectrum at those frequencies will also be deteriorated. For this reason, we proposed a robust distortionless constraint in [11] by modifying this constraint such that the response of the filter at the frequency of interest has a gain which is determined by the signal to noise ratio at that frequency, instead of a unity gain. This process will be the same as weighting the power spectrum value at the frequency of interest based on the ratio of the energy of the signal to the energy of noise at that frequency which makes the MVDR spectrum robust against noise. Therefore, the robust MVDR spectrum for all frequencies will be computed by [11]

(4) $$\begin{array}{c} P_{\text{RMVDR}}\left(\omega \right)=\frac{w{\left(\omega \right)}^{2}}{v^{H}\left(\omega \right)R_{L+1}^{-1}v\left(\omega \right)}, \end{array}$$

where

(5) $$\begin{array}{c} w\left(\omega_{l}\right)=\frac{S\left(\omega_{l}\right)}{N\left(\omega_{l}\right)}, \end{array}$$

where *S*(*ω*_*l*_) and *N*(*ω*_*l*_) are the clean signal and noise at the frequency of interest, *ω*_*l*_, respectively.

Hence, we assign larger weights to the components of the input signal at the frequencies least affected by noise, whereas the others get smaller weights. In [11], employing the experimental findings of psychoacoustics [20, 21], we proposed using the following weighting function with values between zero and one:

(6) $$\begin{array}{c} w_{i}^{2}=1-\exp\left(-\frac{\text{SN}{\text{R}}_{i}}{\gamma_{i}}\right), \end{array}$$

where SNR_*i*_ is the signal to noise ratio computed from the ratio of the energy of noisy signal to noise in the *i*th mel frequency subband and *γ*_*i*_ is the gain that controls the steepness of the weighting function. This weighting function was suggested because using the raw subband signal to noise ratios as the weighting factors did not lead to sufficient recognition accuracies in low SNRs according to experimental results. The following optimum function, which is made up of the difference between two sigmoidal functions, was proposed for *γ*_*i*_ in [11] based on recognition experiments

(7) γi=11+exp⁡(−3(SNRi−0.5)) −11+exp⁡(−3(SNRi−3.5)).

**Figure 1:**
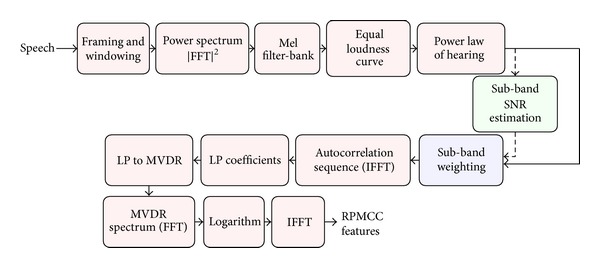
The block diagram for extracting RPMCC features [11]. The subband weighting is applied according to (6) and (7).

The flow diagram for extracting the proposed robust perceptual MVDR-based cepstral coefficients (RPMCC) according to the explained procedure is given in Figure 1. RPMCC features are extracted from the warped power spectrum by incorporating the PLP structure as in [11]. This gives better recognition results because exploiting the perceptual information always improves the speech recognition systems. The equal loudness curve and power law of hearing blocks are according to [3]. We calculate the warped power spectrum by applying the conventional triangular Mel-based filter bank to the FFT-based periodogram. Then the warped MVDR power spectrum is computed from the Mel-warped spectrum after applying weighting to subbands. Then, the cepstral features are calculated by applying IFFT to the Mel-scale MVDR log-spectrum. The Mel-warped spectrum is also known as subband spectrum in the area of speech recognition.

## 3. The Proposed New Front-End Based on Robust MVDR Spectrum of Filtered Autocorrelation Sequence

We assume an additive noise model as follows:

(8) y(m,n)=x(m,n)+v(m,n), 0≤m≤M−1,0≤n≤N−1,

where *x*(*m*, *n*), *y*(*m*, *n*), and *v*(*m*, *n*) represent the clean speech, the noisy speech waveform, and the additive noise, respectively, *m* is the frame index and *n* is the discrete time index within a frame. *M* denotes the number of frames and *N* the number of samples in each frame.

We can have a similar additive equation for autocorrelation of noisy speech, clean speech, and noise on the condition that noise is assumed to be uncorrelated with speech

(9) ryy(m,k)=rxx(m,k)+rvv(m,k), 0≤m≤M−1,0≤k≤N−1,

where *r*_*xx*_(*m*, *k*), *r*_*yy*_(*m*, *k*), and *r*_*vv*_(*m*, *k*) represent the short-time autocorrelation sequences of clean speech, the noisy speech, and the additive noise, respectively, and *k* is the autocorrelation sequence index. If the additive noise is assumed to be stationary, its autocorrelation sequence may be considered to have identical values for all frames. Therefore, we can omit the frame index, *m*, from *r*_*vv*_(*m*, *k*) in (9)

(10) ryy(m,k)=rxx(m,k)+rvv(k), 0≤m≤M−1,0≤k≤N−1.

Hence, we can calculate the differentiation of both sides of (10) with respect to frame index *m*, to find the RAS of noisy and clean speech, which yields [6]

(11) ∂ryy(m,k)∂m=∂rxx(m,k)∂m, 0≤m≤M−1,0≤k≤N−1.

This differentiation can be obtained by an FIR filter on the temporal autocorrelation trajectory. The transfer function of this filter is as follows [6]:

(12) $$\begin{array}{c} H\left(z\right)=\frac{1}{T_{Q}}\sum_{t=-Q}^{Q}tz^{t}, \end{array}$$

where

(13) $$\begin{array}{c} T_{Q}=\sum_{t=-Q}^{Q}t^{2}, \end{array}$$

where (2*Q* + 1) is the frame range for applying the filter.

**Figure 2:**
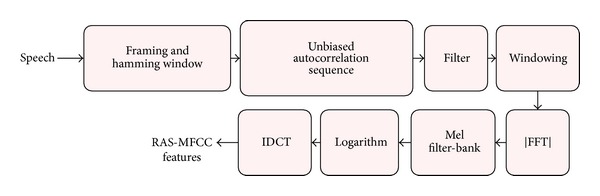
The block diagram for extracting RAS-MFCC features similar to [6]. The windowing block after applying the RAS filter to the autocorrelation sequence is a DDR hamming window to compensate for the variation in dynamic range of the power spectrum of the autocorrelation sequence compared to the corresponding signal's power spectrum. IDCT refers to inverse discrete cosine transform.

The block diagram for extracting the RAS-MFCC features is given in Figure 2. We have changed the strategy proposed in [6] by adding a hamming window at the beginning of the process to make it similar to other speech feature extraction methods. Therefore, a double-dynamic-range (DDR) hamming window is required after applying the RAS filter to find the correct power spectrum. The reason for adding this block is that the power spectrum of an autocorrelation sequence has a dynamic range twice that of the corresponding signal's power spectrum. Hence, to construct an *N*-length DDR hamming window, we perform the following procedure similar to [5]:

- Construct an *N*/2-length Hamming window,
- Calculate its (*N* − 1)-length two-sided (biased) autocorrelation sequence which has a maximum at zeroth lag in the center,
- Pad one zero at the end to make an *N*-length desired window.

**Figure 3:**
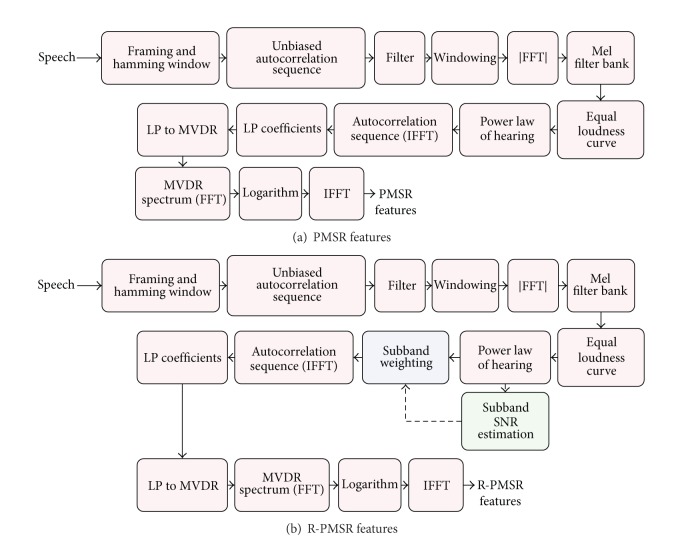
The block diagram for extracting the proposed new front-end based on robust MVDR spectrum of filtered autocorrelation sequence, namely PMSR and R-PMSR features. The filter applied to unbiased autocorrelation sequence is the RAS filter according to (12). The subband weighting for R-PMSR features is calculated according to (14) and (6).

In other words, the RAS of clean speech can be calculated by applying the high-pass filter in (12) to the autocorrelation of noisy speech in the frame range specified by (2*Q* + 1). According to (11), as long as the additive noise is stationary, the RAS of noisy speech will be equal to RAS of clean speech, and thus the effect of noise is removed. However, we often encounter non-stationary additive noise in ASR systems. Therefore, this technique cannot remove the distortions completely and only suppresses DC or slowly varying noise (or stationary noise). In order to reduce the noise residuals which remain after applying RAS filter, we propose finding the MVDR spectrum of this filtered autocorrelation sequence. Moreover, to further suppress the noise effects and thus find more robust features even in low SNRs, we propose using a robust MVDR approach similar to [11] which was explained in Section 2. Therefore, we extract the proposed perceptual MVDR spectrum of relative autocorrelation sequence (PMSR) features from the subband MVDR power spectrum of filtered short-time autocorrelation sequence. The block diagram for extracting the proposed PMSR features is given in Figure 3(a). Also Figure 3(b) illustrates the procedure we proposed for extracting Robust-PMSR (R-PMSR) features. To calculate R-PMSR coefficients, we first pass the short-time autocorrelation sequence through a RAS filter in (12). Then we find the proposed features from the robust perceptual MVDR spectrum of this filtered autocorrelation sequence. The robust perceptual MVDR spectrum is estimated similar to the approach explained in Section 2. However, according to our experiments given in Section 4, the subband SNRs of the signal passed through a RAS filter are increased compared to the case when no filter is applied to the autocorrelation sequence. This happens due to suppression of noise effects by processing the signal with RAS technique. This causes the subband SNRs to be estimated more reliably. Therefore, we propose modifying the subband weighting function suggested in [11]. To this end, we added two free parameters to the steepness controlling gain in (7) to make it more flexible to subband SNR variations

(14) γi=11+exp⁡(−3(SNRi−γ1i)) −11+exp⁡(−3(SNRi−γ2i)),

where

(15) γ1i=0.4+0.11+exp⁡(−(SNRi−1)),γ2i=3+0.51+exp⁡(4(SNRi−1)).

**Figure 4:**
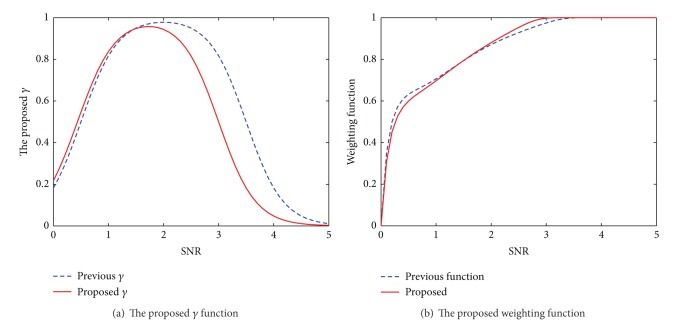
Comparison of the proposed *γ* as the gain controlling the steepness of the weighting function and the resulted weighting function with the one used for extracting robust features when no RAS filtering is used (such as those suggested for RPMCC features). The SNRs in the figures refer to estimated subband SNRs at the output of Mel filter banks followed by equal loudness curve and power law of hearing, as shown in Figure 3(b) and, therefore, are different from SNRs estimated over the whole signal.

When the proposed *γ*_*i*_ in (14) is used as the controlling gain in the subband weighting function in (6), larger weights are assigned to higher SNRs, while lower SNRs get smaller weights. This weighting function makes the algorithm more robust because we encounter less error while estimating the subband SNRs of the warped power spectrum computed from the RAS-filtered autocorrelation sequence compared to the non-filtered case. Therefore, this robust weighting allows the components of the input signal at the frequencies least affected by noise to pass with larger weights, while attenuating those components which are undesirably more affected by noise via assigning smaller weights. In addition, the new proposed function for *γ*_*i*_ makes it more flexible to variations of SNR; that is, it can be more easily tuned to a desired environment. The fixed values used in the proposed functions for *γ*_1_*i*__ and *γ*_2_*i*__ have been selected to increase the speech recognition accuracies. Experimental results given in Section 4 prove the usefulness of this suggested algorithm.  Figure 4 compares the proposed *γ*_*i*_ and the resulted weighting function with those used in [11] for signals not passed through a RAS filter. Therefore, we extract the proposed RPMCC features based on the subband weighting in (7) and (6), while we use (14) and (6) to compute the subband weighting for R-PMSR coefficients.

In order to improve the performance of our algorithm against non-stationary noises, we estimate the noise power spectrum by a simple updating algorithm where the first few nonspeech frames are considered as the initial noise values [22]

(16)  if E[yl(i)]≤βE[Nl(i−1)] then E[Nl(i)]=αE[Nl(i−1)]+(1−α)E[yl(i)] else E[Nl(i)]=E[Nl(i−1)],

where E[*y*_*l*_(*i*)] and E[*N*_*l*_(*i*)] are the estimated energies of the noisy signal and the noise of the *l*th subband in frame *i*, respectively. In addition, we have set *α* to 0.99 and *β* to 2. Furthermore, SNR_*l*_(*i*), which is the signal to noise ratio of the *l*th subband in frame *i*, is calculated as follows:

(17) $$\begin{array}{c} {\text{SNR}}_{l}\left(i\right)=\frac{\text{E}\left[y_{l}\left(i\right)\right]}{\text{E}\left[N_{l}\left(i\right)\right]}. \end{array}$$

For computational purposes, the *L*th order MVDR spectrum is computed using LP coefficients *a*_*k*_  and prediction error variance *P*_*e*_  [10, 19]

(18) $$\begin{array}{c} P_{\text{MVDR}}\left(\omega \right)=\frac{1}{\sum_{-L}^{L}\mu \left(k\right)e^{-j\omega k}}, \end{array}$$

(19) $$\begin{array}{c} \mu \left(k\right)=\{\begin{array}{cc} \frac{1}{P_{e}}\sum_{i=0}^{L-k}\left(L+1-k-2i\right)a_{i}a_{i+k}^{*} & k=0,\ldots ,L\\ \mu^{*}\left(-k\right) & k=-L,\ldots ,-1, \end{array} \end{array}$$

where (2*L* + 1) coefficients of *μ*(*k*) are called the MVDR coefficients and the MVDR spectrum can easily be calculated by an FFT computation according to (18).

## 4. Experimental Results

We have conducted the recognition experiments on Aurora 2 task [23] with clean training scenario. Aurora 2 is a well-known task often used for evaluating the robust speaker-independent speech recognition systems. It has been derived from the TIDigits database, consisting of connected digits spoken by American English talkers, and is downsampled to 8 kHz. It includes two training modes: clean-condition training and multicondition training. In this paper, we only use the clean-condition training set which includes 8440 utterances containing the recordings of 55 male and 55 female adults. All of these signals have already been filtered with G.712 characteristic (a standard telephone line filtering defined by ITU [24]). The test data of Aurora 2 task contain three sets, namely, test sets A, B, and C. 4004 utterances from TIDigits test set data are split into four subsets with 1001 utterances in each. Besides the clean speech signals, one noise type is added to each subset at SNRs of 20 dB, 15 dB, 10 dB, 5 dB, 0 dB, and −5 dB, in order to form the test set. The noises of test set A are suburban train, babble, car, and exhibition hall. In addition, restaurant, street, airport, and train station noises are used to make test set B. Test set C consists of 2 of the 4 subsets. Consequently, while each of the test sets of A and B contains 28028 utterances, test set C is made up of 14014 utterances. Suburban train and street are used as the additive noises in test set C. In this set, speech and noises are filtered with an MIRS characteristic (to simulate the behavior of a telecommunication terminal) before adding the so called noises. Test set C is used to evaluate the performance of ASR systems in case of the presence of both convolutional and additive distortions.

In this paper, we used hidden Markov models (HMMs) to model the digits and pauses using the same topology in [23]. The robustness of the obtained features was evaluated on Aurora 2 task using HTK software [25]. The baseline uses the well-known MFCC features. For extracting all the features, speech was segmented into 25 ms frames with a frame-shift of 10 ms. The Mel filter bank consists of 23 triangular filters. The model order of 15 was used for MVDR-based coefficients which gives the best average recognition accuracy according to our previous experiments. For the features based on filtering the autocorrelation sequence, we used the RAS filter with an order of 2 to get the best recognition results. As usual, we also applied a Juang lifter [2] with a parameter of 22 to cepstral coefficients to further improve the recognition scores for all extracted features. Finally, each frame was represented by a vector consisting of 12 cepstral features augmented by their first and second order derivatives.

**Figure 5:**
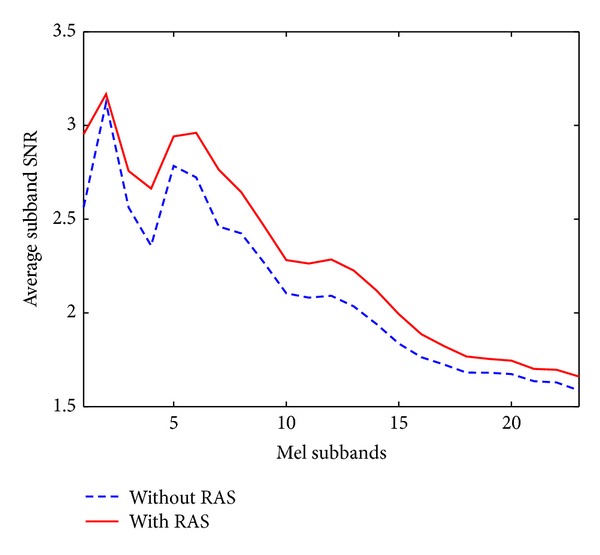
Comparison of the average estimated subband SNRs obtained over the created compact database (containing both clean and noisy data at SNRs from 20 dB to −5 dB) for two cases of using the RAS filter and without it. The SNR in the figure refers to estimated subband SNRs at the output of Mel filter banks followed by equal loudness curve and power law of hearing, as shown in Figure 3(b).

We carried out a set of preliminary experiments to get the idea of modifying *γ*_*i*_ as the steepness controller of the weighting function for the proposed R-PMSR features. To this end, we compared the average subband SNR estimated after applying the RAS filter to the unbiased autocorrelation sequence, with those computed without any filtering (the common Mel-warped spectrum followed by equal loudness curve and power law of hearing). For this reason, we created a compact corpus consisting of 110 Aurora 2 files extracted from the clean training utterances. We have carefully chosen these files such that the resulting compact database includes both single and connected digit utterances. We also added four noises of subway, babble, car, and exhibition at SNRs of 20 dB, 15 dB, 10 dB, 5 dB, 0 dB, and −5 dB following the procedure in [23].  Figure 5 shows the average of estimated subband SNRs obtained over this compact database (containing both clean and noisy data at SNRs from 20 dB to −5 dB) for two cases of using the RAS filter and without it, as explained. Note that the subband SNRs are estimated at the output of Mel filter-banks followed by equal loudness curve and power law of hearing, as shown in Figure 3(b) and, therefore, are different from SNRs or segmental SNRs estimated on the whole signal. According to this figure, applying the RAS filter increases the SNR in all subbands, and therefore the estimated subband SNRs are more reliable. Consequently, we proposed subband weighting based on (6) and (14), as discussed before in Section 3. The fixed values of the functions in (14) and (15) were also tuned using the results of the recognition experiments.

**Figure 6:**
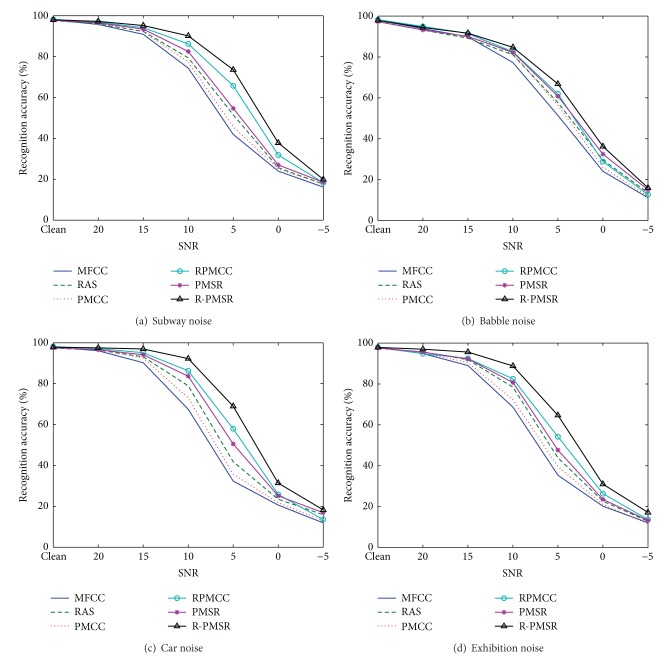
Recognition accuracies for different features in various noise types of test set A of Aurora 2 task. RAS and PMCC refer to RAS-MFCC and perceptual MVDR-based cepstral coefficients, respectively. PMSR and R-PMSR are the proposed robust features.

**Figure 7:**
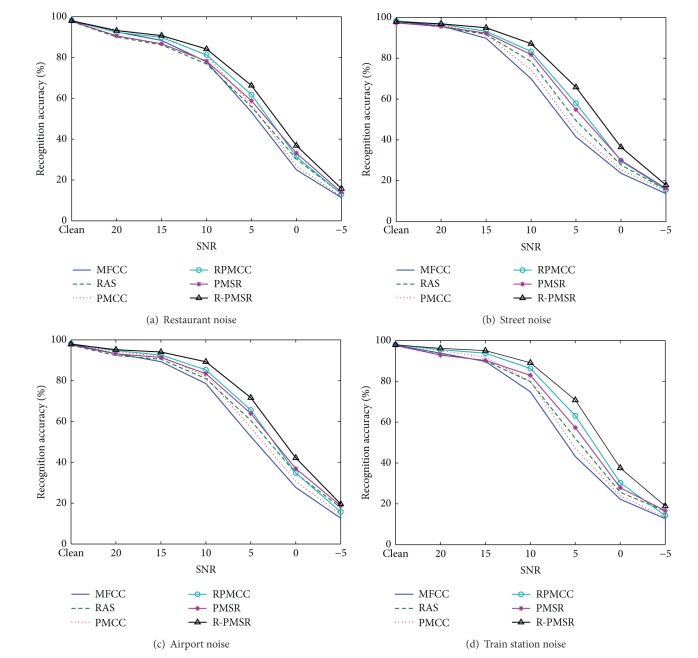
Recognition accuracies for different features in various noise types of test set B of Aurora 2 task. RAS and PMCC refer to RAS-MFCC and perceptual MVDR-based cepstral coefficients, respectively. PMSR and R-PMSR are the proposed robust features.

**Figure 8:**
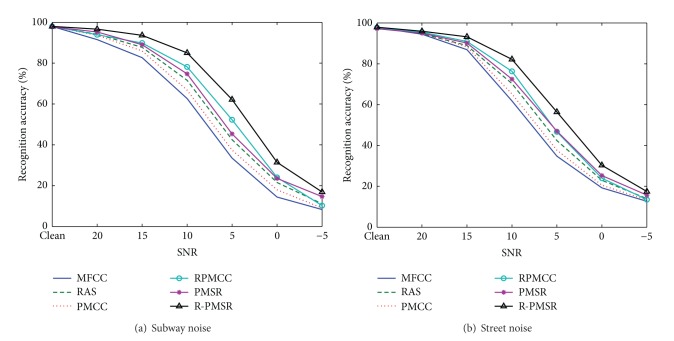
Recognition accuracies for different features in various noise types of test set C of Aurora 2 task. RAS and PMCC refer to RAS-MFCC and perceptual MVDR-based cepstral coefficients, respectively. PMSR and R-PMSR are the proposed robust features.

Table 1 gives the average recognition accuracies of MFCC, RAS-MFCC, PMCC, RPMCC, PMSR, and R-PMSR features over different noise types and SNRs for test sets A, B, and C. PMCC is the MVDR-based features extracted similar to [10, 11]. Figures 6, 7, and 8 show word recognition accuracies for the proposed and baseline features in different types of noises for test sets A, B, and C, respectively.

## 5. Discussion

Table 1 and Figures 6, 7, and 8 clearly show the robustness of the proposed features on three different test sets of Aurora 2 task in different noisy conditions. Even in clean conditions, the performance of the proposed features is not degraded compared to MFCC and PMCC as the baselines. Better accuracy of RAS-MFCC features in comparison with MFCC is the result of filtering the autocorrelation sequence which can reduce DC, low-varying, and stationary noise. In addition, although the recognition accuracies of RAS-MFCC features are slightly lower than MFCCs in clean cases, the proposed R-PMSR features have compensated this drawback. Moreover, better results in noisy cases show that our suggested algorithm is successful in reducing the noise effects remained after applying the RAS filter. R-PMSR coefficients also show much better performance compared to PMSR and RPMCC features in all noisy conditions. This proves the usefulness of the proposed flexible weighting function in (14) and (6) as an advantageous modification to produce a more robust distortionless constraint for MVDR spectrum of filtered autocorrelation sequence. The R-PMSR features lead to a relative improvement of 35.3%, 31.1%, and 34.6% for test sets A, B, and C, respectively, compared to MFCC in the average word error rate (WER). This relative improvement over RAS-MFCC is equal to 26.77%, 24.6%, and 24.8%. Furthermore, PMSR features are more robust than RAS-MFCC in all cases according to the obtained results. This is because of the smaller bias and variance of the estimated perceptual MVDR spectrum used to extract the proposed features. The low bias helps detect low level peaks in the presence of higher ones which preserves the formants of the signals even in low SNRs. Thus, the power spectrum of clean signals will also be extracted more accurately. Moreover, decreasing the variance in estimating the MVDR spectrum makes the undesired fine structure smoother, and hence the extracted features will be more robust against different additive noises.

It is worth mentioning that in this paper we aimed to propose a new robust front-end for speech recognition based on robust MVDR spectrum of filtered short-time autocorrelation sequence of speech signals. Thus we chose MFCC, PMCC, and RAS-MFCC features as the baselines for our experimental comparisons. However, since our suggested technique does not employ any of the complicated enhancement techniques, it can also be used to complement those complex front-ends to make them more robust against environmental noise. Better performance of the proposed R-PMSR features compared to RPMCC proves this claim.

## 6. Conclusion

In this paper, we proposed a new front-end for robust speech recognition. This front-end is based on robust perceptual MVDR spectrum of RAS-filtered autocorrelation sequence. Since we often encounter nonstationary additive noise in ASR, filtering the temporal trajectories of short-time autocorrelation sequences cannot remove the distortions completely. For this reason, we proposed finding the perceptual MVDR spectrum of this filtered autocorrelation sequence to further reduce the noise residuals. This idea led to PMSR features with a better performance than RAS-MFCC in all clean and noisy cases. Moreover, we modified our previously suggested weighting function for RPMCC features to not only adjust it to the new proposed approach but also improve the recognition accuracies in both high and low SNRs. Increasing the subband SNRs of the Mel-warped spectrum which was caused by applying the RAS filter, was the inspiration for suggesting the new flexible weighting function. Therefore, we assigned larger weights to higher SNRs and smaller ones to lower SNRs, compared to RPMCC case. This caused the components of the input signal at the frequencies least affected by noise, to pass with larger weights, while attenuating the noisy and undesired components. In addition, this new proposed function, which is more flexible to variations of SNR, could provide the adaptation to the desired environment more easily. The robustness of the proposed R-PMSR features was achieved by this modified robust distortionless constraint of the MVDR spectral estimation. Acquiring better recognition accuracies in comparison with MFCC, RAS-MFCC, PMCC, and RPMCC features, in most cases, even in clean environments, without employing complex enhancement techniques, is another valuable advantage of the proposed robust feature extraction approach.

## Figures and Tables

**Table 1:** Average recognition accuracies over different noise types and SNRs for test sets A, B, and C and different features.

Feature	Set A	Set B	Set C
MFCC	63.90	66.15	58.21
RAS-MFCC	68.10	69.06	63.70
PMCC	66.25	68.73	60.87
RPMCC	72.36	72.99	67.17
PMSR	70.28	71.11	65.79
R-PMSR	76.64	76.68	72.69
